# Diving Deep into Arrhythmias: Unravelling the Impact of Underwater Environments on Premature Ventricular Complexes in Divers

**DOI:** 10.3390/jcm13175298

**Published:** 2024-09-06

**Authors:** Ivan Ranic, Otakar Jiravsky, Alica Cesnakova Konecna, Bogna Jiravska Godula, Petra Pesova, Jan Chovancik, Radek Neuwirth, Libor Sknouril, Radek Pudil, Jiri Plasek

**Affiliations:** 1 Department of Cardiology, Agel Hospital Trinec-Podlesi, Konska 453, 739 61 Trinec, Czech Republic; ivan.ranic@npo.agel.cz (I.R.);; 2 Research Center for Internal and Cardiovascular Diseases, Faculty of Medicine, University of Ostrava, Syllabova 19, 703 00 Ostrava, Czech Republic; 3 Faculty of Medicine, Masaryk University, Kamenice 735/5, 625 00 Brno, Czech Republic; 4 Faculty of Medicine, Palacky University, Krizovskeho 511/8, 779 00 Olomouc, Czech Republic; 5 1st Department of Internal Medicine—Cardioangiology, Faculty of Medicine in Hradec Kralove, Charles University, Simkova 870, 500 03 Hradec Kralove, Czech Republic; pudilr@lfhk.cuni.cz

**Keywords:** premature ventricular complexes, scuba diving, apnoea diving, arrhythmias in divers, diving physiology, underwater medicine

## Abstract

This review examines the relationship between the physiological demands of diving and premature ventricular complexes (PVCs) in divers. In the general population, some individuals have a greater tendency to experience PVCs, often without awareness or a clear understanding of the triggering factors. With the increasing availability and popularity of both scuba and apnoea diving, more people, including those with a predisposition to PVCs, are engaging in these activities. The underwater environment, with its unique stressors, may increase the risk of arrhythmogenic events, particularly PVCs. Here, we review the prevalence, pathophysiology, and aggravating factors of PVCs in divers, emphasising the need for a comprehensive cardiovascular assessment. Evidence suggests a higher prevalence of PVCs in divers compared with the general population, influenced by factors such as age, dive depth, gas bubbles, cold water immersion, pre-existing cardiovascular diseases, and lifestyle factors. The change in environment during diving could potentially trigger an increased frequency of PVCs, especially in individuals with a pre-existing tendency. We discuss diagnostic strategies, management approaches, and preventive measures for divers with PVCs, noting that although guidelines for athletes can be adapted, individual assessment is crucial. Significant knowledge gaps are identified, highlighting the need for future research to develop evidence-based guidelines and understand the long-term significance of PVCs in divers. This work aims to evaluate potential contributing factors to PVCs in divers and identify individuals who may be at higher risk of experiencing major adverse cardiovascular events (MACEs). This work aims to improve diver safety by promoting collaboration between cardiologists and diving medicine specialists and by identifying key areas for future investigation in this field. This work aims to improve the safety and well-being of divers by understanding the cardiovascular challenges they face, including pressure changes, cold water immersion, and hypoxia. We seek to elucidate the relationship between these challenges and the occurrence of PVCs. By synthesising current evidence, identifying knowledge gaps, and proposing preliminary recommendations, we aim to encourage collaboration between cardiologists and diving medicine specialists to optimise the screening, management, and risk stratification of PVCs in the diving population.

## 1. Introduction

Diving, both as a recreational and professional activity, has gained significant popularity in recent years, exposing individuals to unique physiological challenges. The underwater environment presents a complex interplay of increased ambient pressure, altered gas solubilities, and adaptive physiological responses that can potentially affect the cardiovascular system [1]. Among the various cardiovascular concerns associated with diving, the occurrence of arrhythmias, in particular premature ventricular complexes (PVCs), has attracted attention in recent years.

PVCs are a common type of cardiac arrhythmia characterised by premature depolarisation of the ventricular myocardium, which disrupts the normal rhythm of the heart [2]. While PVCs can occur in individuals with no apparent heart disease, their presence may indicate an underlying cardiac pathology or an increased risk of developing more serious arrhythmias [3]. In the context of diving, the unique physiological stressors encountered underwater may potentially exacerbate or reveal subclinical cardiac conditions, making the study of PVCs in divers a critical area of investigation [4].

It is crucial to distinguish between the two primary types of diving, apnoea (breath-hold) diving and scuba (Self-Contained Underwater Breathing Apparatus) diving, as they involve distinct physiological responses. Apnoea diving activates the mammalian diving reflex, a set of physiological responses aimed at conserving oxygen during prolonged breath-holding [1]. This reflex, more pronounced in apnoea divers, includes bradycardia (slowing of the heart rate), peripheral vasoconstriction, and blood shifting to vital organs [5]. In contrast, scuba diving, where divers breathe compressed air, does not typically elicit the same intensity of diving reflex [6]. Instead, scuba divers face challenges related to breathing compressed gases at depth, including increased partial pressures of oxygen and nitrogen, which can lead to hyperoxia, nitrogen narcosis, and decompression sickness. These fundamental differences in oxygen availability and physiological adaptations between apnoea and scuba diving may have significant implications for cardiovascular function and the occurrence of arrhythmias, including PVCs [7]. Therefore, when discussing the effects of diving on cardiac function, it is essential to specify the type of diving being considered, as the physiological stressors and their potential impacts on the heart can vary considerably.

The number of physiological mechanisms involved in both scuba and apnoea diving activities is complex, and their mutual interactions are crucial for the body’s normal response to diving. Understanding these bodily functions in the context of a higher occurrence of PVCs could lead to strategies for reducing unwanted effects on heart health during diving activities.

PVCs occur with widely varying prevalence in the general population, with estimates ranging from 1% to 4% on standard 12-lead electrocardiograms (ECGs) and up to 75% on 24 h Holter monitoring [8,9]. However, data on the prevalence and significance of PVCs specifically in the diving population remain limited. Given PVCs’ potential impact on diving safety and the need for evidence-based recommendations, it is crucial to implement appropriate screening measures. We suggest that individuals who engage in regular diving activities, particularly those who perform dives below 40 m (which marks the transition from recreational to technical diving), should undergo screening with 24 h, and preferably 48 h, Holter ECG monitoring. This relatively simple yet efficient approach can help identify individuals at higher risk of developing an increased number of PVCs during diving activities.

The pathophysiology of PVCs in divers is of particular interest, as the underwater environment may modulate the arrhythmogenic substrate. Factors such as immersion, exposure to cold water, and the diving reflex may alter autonomic nervous system activity, potentially influencing the development and progression of PVCs [6,10]. In addition, the effects of increased hydrostatic pressure and altered gas solubility on cardiac electrophysiology warrant further investigation [6,10].

Identifying risk factors for PVCs in divers is essential for the development of targeted screening and management strategies. Age, underlying cardiovascular diseases, and diving-specific factors such as depth, duration, and water temperature may contribute to the occurrence of PVCs [11,12]. Understanding the interplay between individual susceptibility and environmental stressors is essential for risk stratification and personalised recommendations.

The diagnosis and monitoring of PVCs in divers requires a tailored approach that takes into account the unique challenges of the underwater environment. Traditional diagnostic tools, such as surface ECGs and Holter monitoring, may not capture the dynamic nature of arrhythmias during diving [13]. Emerging technologies, including underwater ECG monitoring and wearable devices, hold promise for real-time assessments of cardiac function in divers [14,15].

The clinical consequences of PVCs in divers extend beyond the immediate diving context. While the acute impact of PVCs on diving safety is a primary concern, the long-term impact on cardiovascular health should not be overlooked. Frequent or complex PVCs may be associated with an increased risk of developing cardiomyopathy, heart failure, and sudden cardiac death [16,17]. Longitudinal studies are needed to elucidate the prognostic significance of PVCs in divers and to guide follow-up.

The management of PVCs in divers requires a multidisciplinary approach, taking into account the individual’s cardiovascular risk profile, the nature of their diving activities, and the overall impact on their quality of life. Treatment options include pharmacological therapy, lifestyle modification, and interventional procedures such as catheter ablation [18,19,20]. However, the efficacy and safety of these interventions in the specific context of diving remains poorly understood.

The lack of diver-specific guidelines for the management of PVCs highlights the need for evidence-based recommendations. While guidelines for athletes provide a framework for the evaluation and management of arrhythmias in the context of physical activity, the unique demands of diving require a more nuanced approach [21,22].

This review aims to explore the complex relationship between diving and PVCs by examining their prevalence, pathophysiology, and risk factors specifically in the diving population. We will evaluate the impact of unique diving-related stressors, such as immersion, cold water exposure, and the diving reflex, on autonomic nervous system activity and cardiac electrophysiology. This review will assess current diagnostic tools and their limitations in detecting and monitoring PVCs in divers, while also analysing the potential long-term cardiovascular effects of frequent diving and recurrent PVCs. We will review existing management strategies for PVCs in divers and identify areas where diver-specific guidelines are needed. Throughout this analysis, we aim to highlight gaps in knowledge and suggest directions for future research to improve our understanding of PVCs in divers. By synthesising current evidence in these areas, this review aims to improve diver safety, inform clinical decision-making, and contribute to the development of evidence-based guidelines for the management of PVCs in the diving population. Ultimately, our aim is to improve the general understanding of cardiovascular adaptations to extreme environments and support the safe participation of individuals in diving activities.

## 2. Methods

We conducted a comprehensive literature search in PubMed for studies related to PVCs in divers published up to the 1st of May 2024. The search strategy included the following terms: “premature ventricular complexes”, “PVCs”, “divers”, “diving”, “SCUBA diving”, “breath-hold diving”, and “underwater environment”. The search was limited to original research articles published in English and involving adult human subjects.

The inclusion criteria were as follows: (1) studies specifically addressing PVCs related to diving; (2) studies involving adult participants (aged 18 years or older); and (3) studies providing data on the prevalence, characteristics, or risk factors of PVCs in divers. The exclusion criteria were as follows: (1) studies involving children; (2) studies that did not differentiate between atrial and ventricular premature complexes; and (3) non-original research articles, such as reviews, editorials, or case reports.

The quality of the included studies was assessed using the National Institutes of Health (NIH) Quality Assessment Tool for Observational Cohort and Cross-Sectional Studies.

The initial search identified a total of 10 studies. After applying the inclusion and exclusion criteria, 6 studies were considered eligible for inclusion in this review. The included studies were carefully reviewed and relevant data were extracted, including the study design, sample size, diving conditions, prevalence of PVCs, and main outcomes.

Due to the heterogeneity of study designs and outcomes, a formal meta-analysis/systematic review was not feasible. Instead, a narrative synthesis approach was used to summarise and critically appraise the findings of the included studies. The extracted data were organised into thematic sections focusing on the prevalence, pathophysiology, risk factors, diagnostic tools, clinical outcomes, and management strategies related to PVCs in divers. Within each section, the findings were synthesised and any consistencies, discrepancies, and gaps in current knowledge were highlighted.

Limitations of the available literature, such as the small number of studies, the heterogeneity of study populations and diving protocols, and the lack of long-term follow-up data, were also considered and discussed in the context of their potential impact on the interpretation and generalisability of the findings.

## 3. Results

The main section of this review focuses on the various aspects of PVCs in divers, providing a comprehensive overview of the current state of knowledge and understanding. To fully appreciate the significance of PVCs in the diving context, we first provide background information on the nature and characteristics of these arrhythmias. We then review the pathophysiological mechanisms underlying the occurrence of PVCs, with particular emphasis on the unique physiological responses to the underwater environment. The epidemiology of PVCs in the general population and in divers is reviewed, highlighting the potential increased prevalence in the diving community. We also examine the factors that influence the occurrence of PVCs in divers, including age, diving depth, cold water immersion, pre-existing cardiovascular disease, physical fitness, and lifestyle factors. Finally, we discuss management strategies for PVCs in divers, drawing on guidelines for athletes and considering the unique challenges of the underwater environment. By providing a comprehensive review of these topics, we aim to improve the overall understanding of the complex interplay between diving and PVCs, identify gaps in current knowledge, and inform future research and clinical decision-making to promote the health and safety of divers.

### 3.1. Background on Premature Ventricular Complexes

Premature ventricular complexes are a common type of cardiac arrhythmia characterised by premature heartbeats originating in the ventricles. These ectopic beats disrupt the heart’s normal rhythm, often leading to a compensatory pause as the heart’s electrical system resets [2]. While PVCs can occur in healthy people without causing significant harm, their presence can indicate an underlying heart disease or increase the risk of developing more serious heart conditions [3].

PVCs are seen in all age groups but are more common in older adults [23]. Their prevalence varies widely in the general population, with estimates suggesting that up to 75% of healthy adults experience at least one PVC in a 24 h period [8]. The frequency and complexity of PVCs may increase with age and the presence of cardiovascular diseases such as hypertension, coronary artery disease, and heart failure [17].

The clinical significance of PVCs depends on factors such as the frequency, complexity, and underlying cause of the arrhythmia. In the absence of structural heart disease, occasional PVCs are generally considered benign [24]. However, frequent or complex PVCs may be associated with an increased risk of adverse outcomes, including the development of cardiomyopathy, heart failure, and sudden cardiac death [16].

### 3.2. Pathophysiology of PVCs in the General Population and in Divers

The pathophysiology of PVCs involves a complex interplay of different mechanisms that contribute to their occurrence and impact on cardiac function. These mechanisms include triggered activity, enhanced automaticity, re-entry, electrophysiological and structural changes, calcium-handling abnormalities, and the role of sodium channelopathies [2,3,24,25].

In divers, the pathophysiology of PVCs may be further influenced by the unique physiological responses to the underwater environment, which differ significantly between apnoea and scuba diving.

Apnoea Diving:

The diving reflex, predominantly observed in apnoea diving, is triggered by breath-holding and facial cooling. It involves a complex interplay between the parasympathetic and sympathetic nervous systems [26,27,28,29]. During apnoea diving, the body experiences a drop in the partial pressure of oxygen in the arterial blood. In response, the diving reflex redistributes oxygenated blood to vital organs as an oxygen conservation mechanism [27]. This reflex is triggered by the stimulation of arterial receptors and trigeminal nerve endings in the skin, particularly in the nasal vestibule and forehead [28,29].

In apnoea divers, the autonomic nervous system facilitates the body’s response to the diving reflex by simultaneously stimulating the parasympathetic and sympathetic systems. Sympathetic stimulation causes constriction of the peripheral blood vessels and increases the total vascular resistance, redistributing blood to the core of the body and increasing blood pressure [30]. Meanwhile, the parasympathetic nerves slow the heart rate and increase the conduction time of the depolarisation wave from the atria to the ventricles. At the same time, intracardiac sympathetic innervation is activated, which has the opposite positive chronotropic effect [31]. Finally, the vagus nerve exerts a predominant influence on cardiac rhythm, which may be associated with bradycardia [30].

The combination of vagal inhibition of atrioventricular conduction with sympathetically induced enhancement of automaticity and secondary latent pacemakers may increase the risk of PVCs in apnoea divers, particularly when apnoea is preceded by exercise, such as dynamic apnoea swimming or vigorous fin swimming. These types of exercises involve intense physical exertion while breath-holding, which significantly increases sympathetic nervous system activity, elevating the heart rate and blood pressure. This heightened sympathetic tone, combined with the subsequent activation of the diving reflex during apnoea, creates a complex cardiac environment wherein the vagal influence can slow atrioventricular conduction while the sympathetic influence enhances the automaticity of latent pacemakers, increasing the likelihood of PVCs [26].

Scuba Diving:

In contrast, scuba divers breathe compressed air, which maintains a relatively stable partial pressure of oxygen throughout the dive. As a result, the diving reflex is less pronounced in scuba divers [6]. However, scuba diving presents its own set of physiological challenges that may influence PVC occurrence:

Increased ambient pressure: As scuba divers descend, the increased pressure can affect gas solubility in the blood and tissues, potentially influencing cardiac electrophysiology [7].

Breathing resistance: The increased work of breathing at depth due to higher gas density may impact intrathoracic pressures and venous return, potentially affecting cardiac function [32].

Immersion effects: Even without the full diving reflex, water immersion in scuba diving causes central blood volume shifts, which can affect the cardiac preload and potentially trigger arrhythmias [33].

Decompression stress: During ascent, the formation of microbubbles in the bloodstream can potentially irritate the endocardium and trigger PVCs [34].

This complex interplay of physiological responses to the underwater environment, coupled with the inherent mechanisms underlying PVCs, may contribute to an increased susceptibility to arrhythmias in both apnoea and scuba divers [7,35]. The unique environmental factors associated with diving, such as increased ambient pressure, cold water immersion, and the physical exertion required during diving activities, may further exacerbate the risk of PVCs in this population [12,36].

Therefore, a thorough understanding of the pathophysiological mechanisms of PVCs in the context of both apnoea and scuba diving is crucial for the development of appropriate screening, monitoring, and management strategies in order to ensure the safety and well-being of divers with a predisposition to these arrhythmias [37,38].

## 4. Epidemiology

The epidemiology of PVCs has been extensively studied in the general population, providing valuable insights into their prevalence, associated risk factors, and potential impact on health outcomes. However, data on PVCs in divers are limited.

### 4.1. Prevalence of PVCs in the General Population

PVCs are a common finding in both healthy individuals and those with heart diseases. The prevalence of PVCs varies widely depending on the population studied and the methods used for detection. In the Framingham Heart Study, the age-adjusted prevalence of complex or frequent PVCs was 12% among men without clinically evident coronary heart disease and 33% in men with coronary heart disease. The corresponding prevalence in women was 12% and 26%, respectively [23]. In another study using 24 h Holter monitoring, the prevalence of any PVCs was found to be 40–75% in healthy adults, with a higher prevalence in older individuals [9]. Moreover, one meta-analysis of published studies reported that 5.5% of subjects had frequent PVCs, defined as >30 PVCs per hour or >20% of their total heartbeats [39].

### 4.2. Prevalence of PVCs in Divers

Despite the heterogeneity in diving types and study designs, it is valuable to review the available literature on PVCs in diving-related activities. The following paragraph summarises studies that have investigated PVCs in various diving scenarios, including scuba diving, apnoea diving, and face-immersion tests. While these studies are not directly comparable due to their different methodologies and diving types, they collectively provide insights into the occurrence of PVCs across a spectrum of diving-related activities.

Table 1 provides a comprehensive overview of these studies, allowing for a more nuanced comparison of their methodologies and findings.

A scuba diving study by Ali Erdal Gunes (2017) observed ECG abnormalities in professional military divers, with 3 out of 225 divers (1.3%) exhibiting PVCs, which is notably lower than the prevalence typically observed in the general population [40]. Wierzba et al. (2011) enrolled 20 physiology students during a diving reflex study and found that 4 subjects (20%) had PVCs [30]. Malinowski et al. (2023) conducted an apnoea study examining face immersion in cold water (8–10 °C) with 65 participants. They reported rare occurrences of arrhythmias, including three cases of single ventricular extrasystoles during simulated diving tests [41].

A study by Boässon et al. (2019) monitored 17 professional divers using ECGs during a 71 min underwater dive. They reported a total of 10 PVCs, with 7 out of 17 divers (41.2%) experiencing PVCs. Six divers had one PVC each, while one diver had four PVCs [42]. Lemaître et al. (2005) measured heart rate response in trained divers during a breath-holding competition and found that one subject had a couplet of PVCs [43]. Hansel et al. (2009) studied 16 trained breath-hold diving professionals and reported that 9 subjects had either premature atrial complexes (PACs) or PVCs, but the authors did not specify the number of subjects with PVCs alone [35].

Costalat et al. (2021) compared trained breath-holders (BHs) and a group with no subaquatic experience. Both groups underwent face immersion, but only the BH group performed full-body immersion in 26 °C water. While face immersion in 16 °C water was inconclusive, the BH group showed a significant increase in arrhythmogenic events, particularly PVCs, when entering a hypoxemic state (reduction in oxygen saturation (SpO2) by more than 5%) [4].

### 4.3. Why Are Observations of PVCs So Different between Studies?

The current data on the prevalence of PVCs in divers are limited by the small number of studies, the heterogeneity of the study populations, and the varying diving conditions and protocols used. The differences in the reported prevalence of PVCs among the studies may be attributed to factors such as the level of training of the divers, the diving environment (e.g., water temperature, depth), and the duration of the dives.

Wierzba TH et al., 2011 [30], measured ECGs of untrained physiology students as they underwent face immersion into cold water while holding their breath. Lemaître F et al., 2005 [43], held a breath-holding competition among trained divers. Ali Erdal Gunes, 2017 [40], conducted biannual examinations of divers and found no correlation between results after dives. A study by Malinowski et al. [41] examined divers during face immersion in cold water (8 to 10 °C) with the longest apnoea-stimulated diving test. According to Costalat et al., 2021 [4], whole-body submersion in 26 °C water induced a higher prevalence of PVCs in the hypoxemic state than face immersion in 16 °C water. Only Boässon et al., 2019 [42], has studied divers in a long underwater dive with scuba (Self-Contained Underwater Breathing Apparatus) gear: the total dive time was 71 min, with subjects diving in seawater to a depth of 50 m (BM) for 14 min and then having decompression phases at 15 msw for 3 min, at 12 msw for 5 min, at 9 msw for 6 min, at 6 msw for 9 min, and at 3 msw for 33 min. As for the study conducted by Hansel et al., 2009 [35], all of the subjects were trained breath-hold diving professionals, and diving was performed in pools with water temperatures of 27 to 30 °C.

The epidemiological data suggest a potentially higher prevalence of PVCs among divers compared to the general population. In order to better understand this phenomenon, this paper will now explore the various factors that may influence the occurrence of PVCs in divers, including age, diving depth, cold water immersion, pre-existing cardiovascular conditions, physical fitness, and lifestyle factors.

## 5. Factors Influencing PVCs in Divers

**Figure 1:**
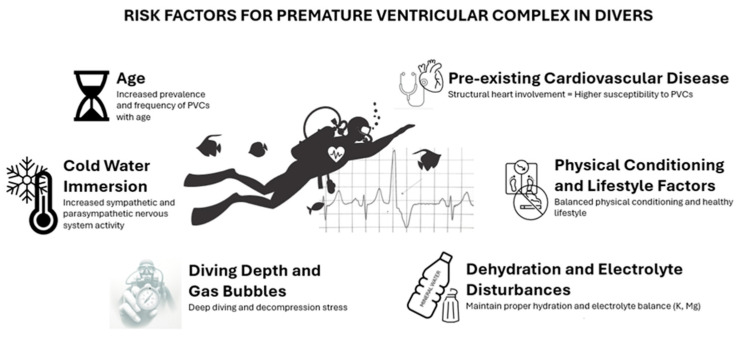
Risk factors for premature ventricular complexes in divers: an overview.

The occurrence of PVCs in divers is likely to be influenced by a complex interplay of individual health factors, the physical demands of diving, and the unique environmental conditions associated with the underwater environment. While the current literature does not provide definitive evidence for specific causative factors, several potential contributors have been identified, as detailed in the following (see also Figure 1).

### 5.1. Age

As with the general population, older divers may be more susceptible to developing PVCs. The prevalence and frequency of PVCs tend to increase with age, possibly due to age-related changes in cardiac structure and function [44]. Older divers should be aware of this potential association and undergo regular cardiovascular evaluations. An age of over 65 years is associated with 80% of all cardiovascular mortality [45].

### 5.2. Diving Depth and Gas Bubbles

In scuba diving, the formation of gas bubbles is a well-recognised phenomenon due to the breathing of compressed air and the associated risk of decompression sickness, especially during rapid ascent. This condition arises from the accumulation of inert gases, such as nitrogen, which become supersaturated in body tissues under high pressure. Upon ascent, the reduction in pressure allows these gases to come out of their solution and form bubbles, potentially leading to serious complications, including arrhythmias.

In contrast, apnoea divers do not breathe compressed air and thus do not accumulate inert gases in the same way. As a result, apnoea divers are generally at a lower risk for decompression sickness since they do not experience the same levels of gas absorption and bubble formation during ascent. However, the repeated and rapid descent and ascent cycles often seen in competitive apnoea diving can still pose a risk, albeit lower, of microbubble formation. These microbubbles, although rare, might still influence cardiac function and potentially lead to arrhythmias, though the mechanism is less pronounced compared to scuba diving. The physiological responses to breath-hold diving, including the diving reflex and associated changes in autonomic nervous system activity, play a more significant role in the occurrence of arrhythmias in apnoea divers [46]. Although the exact mechanisms are not fully understood, the presence of gas bubbles may irritate cardiac tissue, potentially triggering arrhythmias such as PVCs [47].

### 5.3. Cold Water Immersion

Exposure to cold water during diving can lead to an increase in sympathetic nervous system activity, resulting in increased heart rate and blood pressure [48]. This cold-induced cardiovascular response may trigger PVCs in some individuals. Divers should be aware of the potential effects of cold water on their cardiac function and take appropriate precautions, such as using adequate thermal protection [6].

### 5.4. Pre-Existing Cardiovascular Diseases

Divers with an underlying cardiovascular disease, including coronary artery disease, cardiomyopathies, and congenital heart defects, may be more susceptible to developing PVCs [11]. In addition, one study reported that divers experienced a reduction in cardiac output and left ventricular stroke volume after dives, with changes in left ventricular diastolic function suggesting a constrictive effect on the heart, potentially due to the underwater environment [7]. Another study focused on the incidence of arrhythmias and left ventricular hypertrophy in older recreational divers, finding a higher-than-expected prevalence of arrhythmias post dive. This suggests that diving may have acute effects on cardiac rhythm, particularly in individuals with pre-existing cardiovascular conditions or arrhythmias [49]. The physical demands and environmental stressors associated with diving can potentially exacerbate these pre-existing conditions, increasing the likelihood of arrhythmias. Thorough cardiovascular evaluations and individual risk assessments are essential for divers with known cardiac conditions.

### 5.5. Physical Conditioning and Lifestyle Factors

Regular physical activity and maintaining a healthy lifestyle can help reduce the likelihood of PVCs in divers. Studies have shown that individuals with higher levels of physical fitness tend to have a lower prevalence of PVCs [49]. Conversely, factors such as smoking and less physical activity have been associated with a greater frequency of PVCs, indicating that lifestyle factors can also influence the PVC risk [3]. However, excessive training and overexertion should be avoided, as intense physical exertion can also potentially trigger arrhythmias in some cases [50]. Divers should aim for a balanced approach to physical conditioning and allow sufficient recovery time between dives.

### 5.6. Dehydration and Electrolyte Disturbances

Dehydration and electrolyte disturbances that can occur during diving due to immersion diuresis and inadequate fluid intake may potentially contribute to the development of PVCs [51]. Maintaining proper hydration and electrolyte balance is important for cardiovascular stability and may help to reduce the likelihood of arrhythmias in divers.

Figure 1 highlights the risk factors for premature ventricular complexes (PVCs) in divers. These factors include the following: age, which increases the prevalence and frequency of PVCs; pre-existing cardiovascular disease presence, which heightens susceptibility; cold water immersion, which elevates sympathetic and parasympathetic activity; diving depth and gas bubbles, which contribute to decompression stress; physical conditioning and lifestyle factors, emphasising the importance of balanced conditioning and a healthy lifestyle; and dehydration and electrolyte disturbances, underscoring the need for proper hydration and electrolyte balance. Understanding these factors is crucial for conducting comprehensive cardiovascular evaluations of divers and thus for ensuring the safety of divers.

It is important to recognise that the occurrence of PVCs in divers is likely to be multifactorial and may involve complex interactions between individual susceptibility, environmental factors, and diving-related stressors. Further research is needed to better understand the specific mechanisms and to develop evidence-based guidelines for the assessment and management of PVCs in the diving population.

Having identified the potential factors contributing to the occurrence of PVCs in divers, we now shift our focus to the management strategies for these arrhythmias in the diving population. While no specific guidelines exist for managing PVCs in divers, the recommendations for athletes can be adapted to this unique group, taking into account the specific challenges posed by the underwater environment.

## 6. Management Strategies for PVCs in Divers

The management of premature ventricular complexes (PVCs) in divers requires a comprehensive approach that takes into account the unique physiological demands of the underwater environment and the potential impact of PVCs on diving safety. As there are no specific guidelines for managing PVCs in divers, we discuss in the following the recommendations for athletes, as these can be adapted to the diver population.

### 6.1. Guidelines for Athletes with PVCs

#### 6.1.1. Guidelines from the European Society of Cardiology (ESC) (2020)

The ESC guidelines [21] provide recommendations for the management of PVCs in athletes [52]. PVCs are considered a potential marker of underlying heart disease, although there is no absolute threshold for the number of PVCs that warrants further investigation. One study has suggested that asymptomatic athletes with more than 2000 PVCs per day have a 30% chance of having an underlying structural heart disease [53].

The ESC guidelines classify PVCs into common and uncommon locations. Common locations include the right and left ventricular outflow tracts, while uncommon locations include the mitral and tricuspid annuli (especially the posteroseptal region), the His–Purkinje system and intramyocardial foci. Athletes with multiple PVC morphologies originating from the right ventricle should be evaluated for arrhythmogenic cardiomyopathy or sarcoidosis, while those with PVCs originating from the left ventricle should be evaluated for non-ischemic cardiomyopathy.

The guidelines recommend that in athletes with PVCs or non-sustained ventricular tachycardia, possible underlying structural or familial arrhythmogenic conditions should be excluded. The presence of two or more PVCs on a baseline ECG should prompt a comprehensive evaluation, including an investigation into the family history [54]; assessments of the exact number, morphology, and complexity of PVCs using Holter monitoring and 12-lead ECGs; exercise testing; and appropriate imaging. Recommendations for sports participation in athletes with PVCs should be individualised and often require shared decision-making.

#### 6.1.2. Recommendations from the American Heart Association (AHA) and American College of Cardiology (ACC) (2015)

The AHA/ACC scientific statement [22] provides recommendations for the evaluation and management of PVCs in competitive athletes. The statement emphasises the importance of distinguishing between benign PVCs and those that may indicate an underlying pathology. Athletes with PVCs should undergo a minimum level of evaluation before being cleared to compete.

The evaluation should include a thorough history and physical examination, a 12-lead ECG, and an assessment of PVC burden using ambulatory ECG monitoring. Attention should be paid to the presence of transient or chronic cardiac abnormalities and the response of PVCs to exercise. Annual follow-up is recommended for athletes with more than 2000 PVCs per 24 h. Athletes with resting PVCs that increase with exercise should undergo further evaluation, including echocardiography and exercise testing.

### 6.2. Applying Guidelines to Divers with PVCs

As stated earlier, although there are no specific guidelines for the management of PVCs in divers, the recommendations for athletes can be adapted to this population. Divers, like athletes, are subject to physiological stresses that can potentially exacerbate underlying cardiac diseases. However, the underwater environment and the specific demands of diving should be considered when applying these guidelines.

Divers with PVCs should undergo a comprehensive cardiovascular evaluation as recommended for athletes. The evaluation should include assessments of PVC burden, PVC morphology, and the PVCs’ response to exercise, as well as screening for underlying structural heart disease. The decision to allow a diver with PVCs to continue diving should be based on an individual risk assessment, taking into account the severity of the PVCs, the presence of any underlying cardiac diseases, and the specific diving conditions that the individual is subjected to during their dives.

Preventive strategies such as regular medical check-ups, a healthy diet, avoidance of hypoxia, and education on safe diving practices are important for all divers, especially those with PVCs. These strategies can help to identify potential risk factors, minimise the risk of arrhythmias, and promote overall cardiovascular health in the diving population.

Shared decision-making between individual divers, cardiologists, and dive medicine specialists is essential for determining the most appropriate management strategy. Factors such as the depth and duration of dives, water temperature, and the presence of other risk factors should be considered when making recommendations for diving activities.

The distinctions between apnoea and scuba diving have significant implications for both the occurrence and management of PVCs in divers. In apnoea diving, the pronounced diving reflex and associated bradycardia, coupled with periods of hypoxia, may create a unique electrophysiological environment conducive to PVC formation. The rapid alternation between sympathetic activation during exercise and parasympathetic dominance during the dive may increase an individual’s susceptibility to arrhythmias. Management strategies for apnoea divers with PVCs should focus on optimising dive times, ensuring adequate recovery between dives, and potentially implementing breath-hold training techniques to enhance their ability to conserve oxygen during dives.

In contrast, scuba diving presents different challenges. While the diving reflex is less pronounced, the increased ambient pressure, altered gas partial pressures, and potential for bubble formation during decompression introduce distinct arrhythmogenic risks. Management approaches for scuba divers with PVCs should emphasise proper ascent techniques, adherence to decompression protocols, and considerations of dive profiles that minimise rapid pressure changes. Additionally, the use of enriched air/nitrox might be considered to reduce nitrogen loading, potentially mitigating some of the physiological stresses associated with breathing compressed gas.

For both types of diving, comprehensive cardiovascular screening, including exercise stress testing and consideration of underwater conditions, is crucial. The development of specific guidelines for each diving modality, taking into account these physiological differences, would greatly enhance the safety and management of divers with PVCs. Future research should aim to elucidate the specific mechanisms by which each type of diving influences PVC occurrence, enabling more targeted preventive and management strategies.

Medical therapy and lifestyle modifications, including beta-blockers, anti-arrhythmic drugs, catheter ablation, avoidance of stimulants, stress reduction, regular exercise, and adequate hydration, may be applicable to divers with PVCs, depending on the individual’s specific circumstances and the underlying cause of their arrhythmia.

## 7. Conclusions

Diving exposes individuals to unique physiological challenges that may exacerbate or trigger arrhythmias, particularly PVCs. Current evidence suggests a complex interplay between individual susceptibility, environmental factors, and diving-related stressors in the pathogenesis of PVCs. However, significant knowledge gaps remain regarding the precise mechanisms and long-term effects of PVCs in divers, as well as regarding optimal strategies for PVC management in divers.

This review highlights the need for comprehensive cardiovascular evaluations and tailored diagnostic and monitoring approaches for divers with PVCs. The lack of diving-specific guidelines pertaining to cardiac health underlines the importance of collaborative efforts between diving specialists, cardiologists, and exercise physiologists to establish evidence-based recommendations that prioritise diver safety.

Future research should focus on elucidating the complex relationships between diving and PVCs, with an emphasis on prospective studies, standardised protocols, and long-term follow-up monitoring. The potential utility of emerging technologies for real-time cardiac monitoring during diving activities should also be explored.

Furthermore, this review highlights the need for more focused research on the differences between scuba and apnoea diving in influencing heart rhythm disturbances. Future studies should aim to elucidate the specific mechanisms by which each type of diving affects cardiac function and arrhythmia risk. Such research could provide valuable insights into the pathophysiology of PVCs in divers and potentially lead to more tailored prevention and management strategies. Comparative studies examining the acute and chronic effects of scuba diving versus apnoea diving on cardiac electrophysiology would be particularly valuable in addressing this knowledge gap.

Ultimately, the knowledge gained from studying PVCs in divers may have broader implications for understanding cardiovascular adaptations to extreme environments and developing targeted interventions for individuals at risk of arrhythmias. Continued research and collaboration in this area is essential for optimising cardiovascular health in divers and supporting their safe participation in this challenging and rewarding activity.

## Figures and Tables

**Table 1:** Summary of studies on the prevalence and characteristics of premature ventricular contractions in divers.

Authors	Total Subjects	PVCs (N, %)	Participants (Male/ Female)	Age (Years)	BMI (kg/m^2^)	Water Temperature (°C)	Tank	Additional Insights	Diving/ Immersion Type
Ali Erdal Gunes, 2017 [40]	225	3 (1.3%)	204/21	26.5 ± 5.7	23.4 ± 2.8	N/A	N/A	Study focusing on ECG changes in professional divers. Findings: Most common ECG abnormality was incomplete right bundle branch block (IRBBB).	Scuba diving
Wierzba TH et al., 2011 [30]	20	4 (20%)	20/0	18.9 ± 0.9	22.8 ± 2.9	8–10	N/A	Study on the effects of immersion and cold water on arrhythmias. Findings: 20% of subjects showed PVCs, significantly higher than other studies.	Face immersion
Malinowski K et al., 2023 [41]	65	5 (7.7%)	28/37	21.13	21.49	8–10	N/A	Comprehensive study including both male and female divers. Findings: Lower percentage of PVCs compared to other studies, emphasising gender-based differences.	Face immersion
Boässon MP et al., 2009 [42]	17	7 (41.2%)	17/0	39.5 ± 7.0	N/A	18.5 ± 0.79	15 L oxygen tank, 200 bar	Prospective study using Holter monitoring. Findings: Significant prolongation of PR interval, decrease in QRS duration, and a high incidence of PVCs.	Scuba diving
Lemaître F et al., 2005 [43]	16	1 (6.25%)	16/0	25.6 ± 6.9	N/A	27–28	N/A	Analysis of cardiac responses during breath-holding. Findings: Bradycardia and arrhythmias observed, with a focus on the physiological adaptations in well-trained divers.	Apnoea diving
Costalat et al., 2021; [4]	9	2 (22.2%)	9/0	38.8 ± 8.6	25.2 ± 4.4	15.7 ± 1.7	N/A	Study on breath-hold divers. Findings: Moderate percentage of PVCs with a focus on physiological stress during breath-hold dives.	Face immersion
Costalat et al., 2021; [4]	9	6 (66.7%)	9/0	38.8 ± 8.6	25.2 ± 4.4	26	N/A	Comparison with the above “BH, FI” study. Findings: High incidence of PVCs, possibly due to different physiological responses or stress levels in divers.	Whole-body immersion

Notes: This table summarises the findings from various studies on the prevalence and characteristics of PVCs in divers. The table includes information on the total number of subjects, the number and percentage of subjects with PVCs, participant demographics, the water temperature during the dives, and additional insights from each study. The studies span different types of diving activities and conditions, providing a comprehensive overview of the factors influencing PVC occurrence in divers. Abbreviations: PVCs—premature ventricular contractions; N/A—not available; BH—breath-hold; FI—face immersion; WBI—whole-body immersion; BMI—Body Mass Index.
